# Effects of lorazepam on saccadic eye movements – evidence from prosaccade and free viewing tasks

**DOI:** 10.1007/s00213-024-06672-z

**Published:** 2024-09-03

**Authors:** Philine M. Baumert, Kaja Faßbender, Maximilian W. M. Wintergerst, Jan H. Terheyden, Behrem Aslan, Tom Foulsham, Wolf Harmening, Ulrich Ettinger

**Affiliations:** 1 Department of Psychology, University of Bonn, Kaiser-Karl-Ring 9, 53111 Bonn, Germany; 2 Department of Ophthalmology, University of Bonn, Bonn, Germany; 3 Department of Psychiatry and Psychotherapy, University of Bonn, Bonn, Germany; 4 Department of Psychology, University of Essex, Colchester, UK; 5 Augenzentrum Grischun, Chur, Switzerland

**Keywords:** Lorazepam, Benzodiazepine, Saccadic eye movements, Biomarker, Prosaccades, Free viewing, Peak velocity

## Abstract

**Rationale:**

Peak velocities of saccadic eye movements are reduced after benzodiazepine administration. Even though this is an established effect, past research has only examined it in horizontal prosaccade tasks.

**Objectives:**

The spectrum of saccadic eye movements, however, is much larger. Therefore, we aimed to make a first attempt at filling this research gap by testing benzodiazepine effects on saccades under different experimental task conditions.

**Methods:**

1 mg lorazepam or placebo was administered (within-subjects, double-blind, in randomised order) to *n* = 30 healthy adults. Participants performed an extended version of the prosaccade task, including vertical saccade directions and different stimulus eccentricities, as well as a free viewing task.

**Results:**

Results from the prosaccade task confirmed established effects of benzodiazepines as well as saccade direction on saccadic parameters but additionally showed that the drug effect on peak velocity was independent of saccade direction. Remarkably, in the free viewing task peak velocities as well as other saccade parameters were unaffected by lorazepam. Furthermore, exploration patterns during free viewing did not change under lorazepam.

**Conclusions:**

Overall, our findings further consolidate the peak velocity of prosaccades as a biomarker of sedation. Additionally, we suggest that sedative effects of low doses of benzodiazepines may be compensated in tasks that more closely resemble natural eye movement behaviour, possibly due to the lack of time constraints or via neurophysiological processes related to volition.

**Supplementary Information:**

The online version contains supplementary material available at 10.1007/s00213-024-06672-z.

Benzodiazepines are amongst the most commonly prescribed anxiolytics. They exert their effects through nonspecific inhibition of central nervous system activity, via positive allosteric modulation of the GABA_A_ receptor in the γ-aminobutyric-acid (GABA) system. Whilst clinically effective, benzodiazepines have side effects, such as sedation (Knoflach and Bertrand 2021; Shader and Greenblatt 1993; Uusi-Oukari and Korpi 2010). Recent research has attempted to determine how anxiolytic and sedative effects arise in order to develop new drugs without sedative action (Möhler et al. 2002; Uusi-Oukari and Korpi 2010).

One highly sensitive measure of sedative drug effects is the peak velocity of saccadic eye movements (Chen et al. 2014). Saccades are rapid eye movements that bring the image of an object of interest onto the fovea. The peak (or maximal) velocity of horizontal saccades is slowed significantly after benzodiazepine administration – a finding that is so reliable that the measure is considered a biomarker of sedation (Chen et al. 2012; Connell and Baxendale 2011; De Visser et al. 2003). Even though the effect is well replicated, previous research has focused almost exclusively on horizontal, visually-guided saccades in prosaccade tasks (De Visser et al. 2003).

However, the saccadic repertoire is much greater (Leigh and Zee 2015), including saccades in other directions in space and in response to different types of stimuli or tasks. These task differences are mirrored on a neurophysiological level: Burst neurons in the paramedian pontine reticular formation (PPRF) provide commands for horizontal saccades (Horn 2006; Yoshida et al. 1982), whereas burst neurons in the rostral interstitial nucleus of the medial longitudinal fasciculus (riMLF) enable premotor commands for vertical saccades (Horn and Büttner-Ennever 1998; King and Fuchs 1979). Both receive excitatory input from the superior colliculus that influences peak velocity and amplitude (Fuchs et al. 1985; Schiller et al. 1979; Scudder 1988). Benzodiazepines impede these excitatory signals as they amplify effects of GABA on the GABA_A_ receptor which leads to hyperpolarisation of cells, thus reducing the intensity of excitatory input that burst neurons in the PPRF and riMLF receive and slowing saccadic velocity as a result (Fuchs et al. 1985; Hikosaka and Wurtz 1985; Schiller et al. 1979). Notably, vertical saccades have systematically slower velocities than horizontal ones (Bahill and Stark 1975; Becker and Jürgens 1990; Collewijn et al. 1988), presumably because burst neurons in the riMLF generally fire with lesser intensity (Fuchs et al. 1985). Consequently, it stands to reason that benzodiazepines might affect vertical saccades differently than horizontal ones.

Therefore, the present study aimed to examine benzodiazepine effects on saccadic peak velocity by employing an expanded prosaccade task including both vertical and horizontal saccades. Further, Ettinger et al. (2018) found the effect of the benzodiazepine lorazepam on peak velocity to be larger for peripheral stimuli closer to the centre of the screen than for stimuli with larger eccentricities. To replicate this finding, the current prosaccade task also included two different stimulus eccentricities (near, far) for each saccade direction (horizontal, vertical).

Aside from reducing peak velocity, benzodiazepines have been shown to increase saccadic latencies (Bey et al. 2021; Green et al. 2000; Masson et al. 2000) and decrease saccadic amplitudes (Ettinger et al. 2018; Masson et al. 2000). Although replicable, these effects are not as pronounced or consistent as the effect on peak velocity (Bey et al. 2021; de Haas et al. 2007; Ettinger et al. 2018; Lynch et al. 1997). To produce a coherent picture of benzodiazepine effects on saccadic eye movements, latency and amplitude were also examined.

Experimental prosaccade tasks are not reflective of saccadic behaviour in everyday life: The amount of visual information to be scanned, filtered, and processed is much lower in such tasks than what typically occurs under natural circumstances. Consequently, benzodiazepine effects on saccades in a prosaccade task may differ from what patients taking these anxiolytics experience. Saccades generated when people navigate or explore their environment have a greater voluntary quality than prosaccades as they require higher levels of control (Foulsham 2015), are subject to top-down influences (Helo et al. 2014; Henderson 2007), and differ on a neurophysiological level (Leigh and Zee 2015; Pierce et al. 2019).

Therefore, a free viewing paradigm was employed to elicit the generation of more ‘natural’ saccades and study their modulation by benzodiazepines. As this has not, to our knowledge, been studied before, research questions regarding the free viewing task^1^ were exploratory. Specifically, we examined whether benzodiazepine effects on saccadic peak velocity would also be found in a free viewing task. Unlike the highly constrained prosaccade task, participants in this task choose to make saccades of many different directions and sizes. Thus, saccadic amplitude and frequency were also assessed to comprehensively evaluate benzodiazepine effects.

A large literature has examined the bottom-up and top-down components of attentional and saccadic control in free viewing tasks (Foulsham 2019; Itti and Koch 2001; Peters et al. 2005). Saccade generation involves distinct neurophysiology according to whether targets are selected in a stimulus-driven or top-down fashion. If benzodiazepines selectively affect some components more than others, the correlation between fixations and stimulus saliency might be affected. To explore this, the distribution of fixations was compared to the output of the Graph-Based Visual Saliency (GBVS) model, a bottom-up model predicting human fixations based on visual image features. The GBVS model achieves higher accuracy than classical algorithms by using a dissimilarity metric (Harel et al. 2006; Judd et al. 2012). It is, like the Itti and Koch (2001) model, based on the theoretical approach to feature-based saliency models by Koch and Ullman (1985). This analysis should also reveal changes in fixation distribution (*what* people look at) beyond the expected change to saccade metrics.

## Method

### Sample

Thirty healthy, non-smoking students completed the study. Sample size was based on an a priori G*Power analysis (V 3.1; Faul et al. 2007), aiming for > 85% power with an effect of *d* = 0.5 and an alpha-level of 0.05. The sample was balanced for gender (female, male). Recruitment took place via local and online advertisements in and around the city of Bonn, Germany. The study was approved by the ethics committee of the Faculty of Medicine at the University of Bonn (Lfd. Nr. 240/19), carried out in compliance with the Declaration of Helsinki, and preregistered at OSF (Open Science Framework; 10.17605/OSF.IO/GQBWN) prior to data collection.

Participants first filled in an online screening questionnaire. If they fit the basic inclusion criteria (aged 18 to 40 years, healthy, right-handed, non-smoking, and normal or corrected to normal vision) they were invited to a second, more detailed in-person-screening. Exclusion criteria were medication consumption (except oral contraceptives in women), current physical, neurological or psychiatric diagnosis, blood pressure < 100/60 or > 140/90, resting pulse < 60 or > 100 beats per minute, body mass index (BMI) < 18 or > 29 for men or < 19 or > 30 for women, nicotine consumption (more than 10 cigarettes in lifetime), positive drug (nal von minden Drugscreen ® Multi 5N test) or alcohol (ACE ALCOSCAN AL5500 plus Breathalyzer) test, earlier consumption of lorazepam or other benzodiazepines (lifetime), known allergic reactions to medications, and, for women, a positive pregnancy test (Cleartest®Diagnostik HCG), breastfeeding or not using effective contraceptives for the duration of at least one cycle. After completion of the experimental sessions, participants were compensated with either 100 € or course credits (for psychology students).

### Design and procedure

A crossover, double-blind, randomised, and placebo-controlled design was employed.

Each participant took part in two 4 h long experimental sessions, exactly one week apart at the same time of day (either 8:30 am or 1:00 pm). At the beginning of each session, participants confirmed their consent and current health, took another alcohol test and, for women, another pregnancy test. Subsequently, they were administered either 1 mg lorazepam (Tavor™, Pfizer) or placebo (mannitol). Drugs were taken orally with a glass of water, had no odour, and were encapsulated indistinguishably. Following drug administration, participants waited for 1.45 h to start experimental tasks (Saari et al. 2011).

Experimental tasks consisted of an oculomotor and cognitive test battery lasting about 70 min in total. Oculomotor task order was randomised between participants but kept constant between sessions. After completion of tasks, an online questionnaire comprising the visual analogue scales (VAS; Bond and Lader 1974), the NASA-TLX task load index (NASA-TLX; Hart and Staveland 1988), and an item having participants guess whether they had received lorazepam or placebo that day was assessed. The present paper focusses on the prosaccade and free viewing tasks, which were carried out between 105 and 160 min after drug administration. As assessments took place during the Covid-19 pandemic, both participants and researchers wore facemasks during the experimental sessions.

### Eye-tracking

Eye movements were measured using a video-based, combined pupil and corneal reflection system (EyeLink 1000, SR Research Ltd., Toronto, ON, Canada; EyeLink host software version 4.594) at a sampling frequency of 1000 Hz. Assessments were carried out in a darkened room. Horizontal and vertical eye positions were recorded binocularly, although only right eye data were examined here. Head movements were constrained by a chin and forehead rest placed 220 cm from the monitor. The monitor was a Sony 55XE8505, 55″ flat screen (121 cm width x 68 cm height, 138.8 cm diagonal) with a resolution of 3840 × 2160 px (30.75 × 17.57° visual angle, 122 pixels per degree) and a refresh rate of 59 Hz. Oculomotor tasks were written in ExperimentBuilder (Version 2.3.38, SR Research Ltd., Mississauga, Canada) and had a black (r, g, b: 0, 0, 0) background.

Before the first oculomotor task, a 9-point calibration (grid of 3 × 3 round white stimuli with a black dot in the middle, presented in random order) and subsequent validation was performed. Routines and default settings provided by the manufacturer were employed. Participants were excluded if their validation did not reach an average error below 1° visual angle or if the validation was not graded “good”. The procedure was repeated before a task if participants had moved between tasks. Empirical accuracy and precision (Hutton 2019) are reported in the supplementary material.

### Tasks

In the prosaccade task (for an exemplary figure of the task sequence, see supplementary material, Fig. S1), the stimulus was a white circle (255, 255, 255; diameter: 15 px or 0.12°; stroke width: 5 px or 0.04°). The circle was first presented in the centre of the screen for 1000 to 2000 ms, varied at random, and participants were asked to fixate its centre. Then, it appeared in the periphery for 1000 ms and participants were asked to look at its centre as fast and accurately as possible. There were eight different peripheral locations: up, down, left, or right from the centre of the screen at two different eccentricities, near (4°) or far (8°). In total, participants performed 120 trials each session, 15 per peripheral location. The experimental task was preceded by 8 practice trials.

For the free viewing task (for an exemplary figure of the task sequence, see supplementary material, Fig. S2), emotionally neutral pictures from the international affective picture system (IAPS; Lang et al. 2008) were chosen by the following procedure. First, stimuli with many image details were selected. Second, images that looked obviously dated were avoided. Third, stimuli were retained if their valence, arousal, and dominance according to the IAPS manual (Lang et al. 2008) was between 3 and 7. In each assessment session, 20 pictures were shown, with four pictures taken from each of the categories people, animals, objects, plants, and nature scenes (for IAPS picture numbers, see supplementary materials). Selected pictures were divided to provide two matched versions of the task. There were no significant differences in mean or standard deviation of valence, arousal or dominance between the two task versions (all *p* > 0.23). Each participant viewed one picture set in the first session and the other in the second session, randomly determined, such that across all participants the picture sets were equally present in each drug condition.

Each picture was shown for 5 s and participants were instructed to memorise them for a later memory test in order to encourage thorough viewing. In reality, memory was never formally tested but after the first assessment, participants were asked some questions regarding the pictures to ensure an incentive to perform adequately during the second assessment. Pictures were displayed in the centre of the screen and had a size of 1024 × 768 px (horizontal x vertical). Before each picture, a fixation stimulus was shown for 1000 ms. Its properties matched the stimulus in the prosaccade task.

### Data processing

To determine eye-tracking events, data were preprocessed in accordance with the EyeLink host software’s default settings.^2^ Eye-tracking data were examined visually concerning data quality and data reports were generated using the EyeLink DataViewer (version 4.2, SR Research Ltd., Mississauga, Canada). Data were then further preprocessed using R (R Core Team 2021) as follows. Subjects who only attended one session were excluded from all analyses. In the prosaccade task, only the first saccade that occurred after peripheral stimulus presentation was included per trial. Of those, saccades that contained blinks, went in the wrong direction, started more than 100 px from the centre of the display in any direction, had an amplitude < 1° or a latency < 70 ms or > 1000 ms were excluded from subsequent analyses. Participants with fewer than 5 valid trials per factor level for ANOVA calculations were also excluded. In the free viewing task, saccades were excluded from analyses if they contained blinks or had an amplitude < 1°. For the GBVS analysis, fixations in the free viewing task were re-calibrated as visual inspection showed that almost all fixations were dislocated down and to the left. To adjust fixations, for the initial central fixation in each trial, the mean distance between the fixation stimulus and recorded fixations weighted for the duration of the fixation were calculated per subject and then added to the recorded fixations during the free viewing part of the same trial. Only fixations with a duration > 100 ms were included in the final analysis.

To calculate Cronbach’s α, a random sample of values was drawn per participant and task for each dependent variable and configuration of independent variables. A minimum of 10 values per participant, dependent variable, and combination of independent variables was set. If there were less than 10 values available, the participant was excluded from that sample. If there were more than 10 values, the number of values drawn was equal to the lowest number of available values present. The number of participants included as well as values extracted per task and configuration of independent variables can be found in the supplementary material.

### Statistical analyses

Data were analysed using R (R Core Team 2021) and the following R packages: *apaTables* (Stanley 2021), *dplyr* (Wickham et al. 2023), *ez* (Lawrence 2016), *ggplot2* (Wickham 2016), *mice* (van Buuren and Groothuis-Oudshoorn 2011), *patchwork* (Pedersen 2023), *rstatix* (Kassambara 2023), and *weights* (Pasek 2021). Within-subjects ANOVA with the factors drug (lorazepam, placebo) and saccade direction (horizontal, vertical) were performed separately for the prosaccade and the free viewing task. For all analyses, group means of all dependent variables were used and *p* < 0.05 was applied as a significance criterion.

In the prosaccade task, the additional factor of stimulus eccentricity (near, far) was included and peak velocity, peak velocity corrected for saccade amplitude, amplitude gain (i.e., the relationship of measured saccadic amplitudes to actual stimulus position),^3^ latency, and data loss^4^ were analysed as dependent variables. Dependent variables in the free viewing task were saccadic peak velocity, peak velocity corrected for amplitude, saccade amplitude, saccade frequency, and data loss. Further, within-subjects ANOVA with the factor drug (lorazepam, placebo) were performed for VAS and NASA-TLX measures. Effect sizes were reported as partial eta squared (η^2^_*p*_) and its confidence interval (CI; Cohen 1973). Greenhouse–Geisser correction was applied in case Mauchly’s test indicated a violation of sphericity. In further explanation of significant effects, Bonferroni-corrected post-hoc *t*-tests were carried out and effect size *d*_*AV*_ is reported (Lakens 2013).

**Fig. 1:**
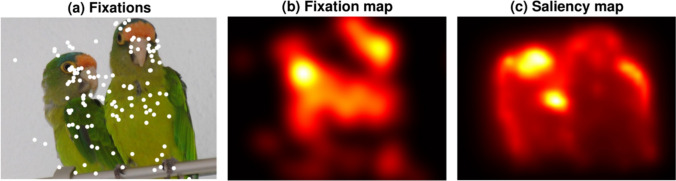
Exemplary fixation and GBVS maps. *Note.* **a** exemplary fixation data from 3 subjects who performed free viewing on the image for 3 trials à 5 s each. **b** a heat map created from fixations in a). **c** GBVS map for the given same image

To compare fixations with visual saliency, a saliency map was created for each image using the GBVS algorithm provided by Harel et al. (2006) and implemented in MATLAB (The MathWorks Inc, 2022, version 9.9.0; R2020b). The fixations from all participants in each drug condition were combined, per image, to produce fixations maps. These maps show the relative frequency of fixations on different parts of the image in the lorazepam and placebo conditions (for an example, see Fig. 1). Saliency maps and fixation maps were then correlated to measure the correspondence between salient features and fixation locations. This was implemented using code from the MIT/Tuebingen saliency benchmark (http://saliency.tuebingen.ai) and fixation maps were generated with a low-pass filter of 8 cycles per image (for more information on metrics, see Bylinskii et al. 2019). The set of correlations between drug conditions was compared using a dependent-samples *t*-test.

To assess possible influences of the within-subjects design, order of drug administration was included as a between-subjects factor in additional analyses for both tasks and all dependent variables.

## Results

In total, 30 (15 female) participants (mean age = 22.90, *SD* = 3.35) concluded both assessments. Due to drop out of four participants because of the corona virus before completing the second assessment, the order of drug administration was not evenly distributed: 17 participants were administered placebo first and lorazepam second, whereas 13 participants underwent the opposite order. Order of drug administration did not alter the main results for either task. Full results for analyses including the order of drug administration as a factor can be found in the supplementary material (Table S2 a), S2 b), and S3 for the prosaccade task and S4 for the free-viewing task).

### Prosaccade task

**Fig. 2:**
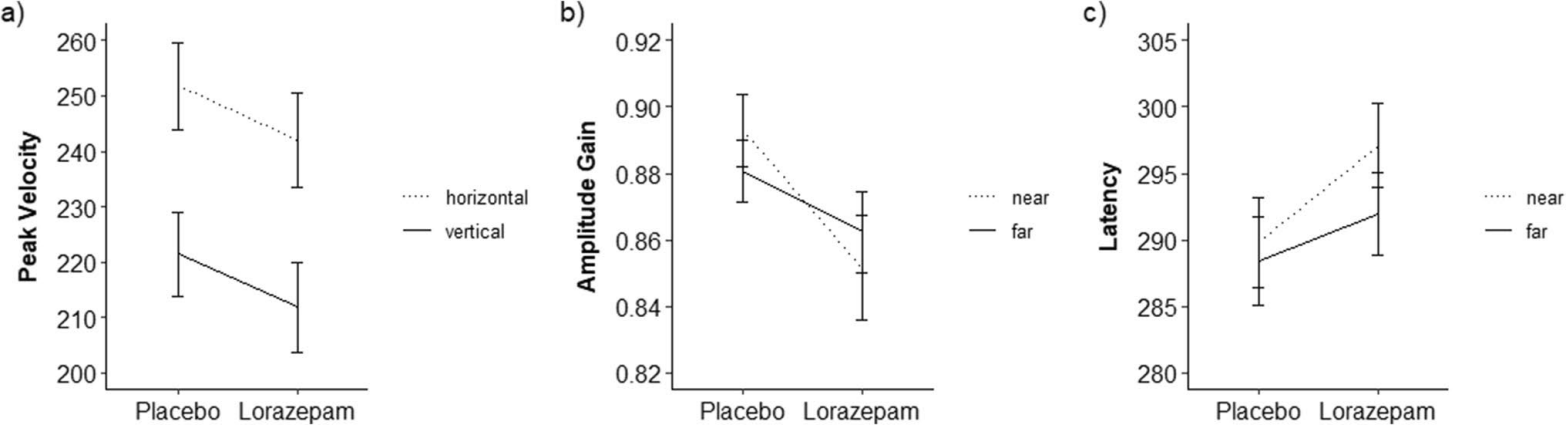
Effects of drug condition in the prosaccade task. *Note.* Error bars indicate standard error of the mean. **a** shows the effect of drug condition and saccade direction on saccadic peak velocity in °/s, **b** the effect of drug condition and stimulus eccentricity on amplitude gain, and **c** the effect of drug condition and stimulus eccentricity on saccadic latency in ms

One participant was excluded due to fewer than five valid trials per factor level for the ANOVA calculations, resulting in *n* = 29. Descriptive results can be found in Table 1. A main effect of lorazepam (Fig. 2a) on peak velocity (*F*_(1, 28)_ = 8.01, *p* = 0.008, $\eta$_p_^2^ = 0.223, CI [0.016,0.445]) replicated the established finding that benzodiazepines slow saccadic peak velocity in comparison to placebo. Additionally, lorazepam significantly reduced amplitude gain (*F*_(1, 28)_ = 6.63, *p* = 0.016, $\eta$_p_^2^ = 0.191, CI [0.006,0.416]) and increased latency (*F*_(1, 28)_ = 6.73, *p* = 0.015, $\eta$_p_^2^ = 0.194, CI [0.007,0.419]) compared to placebo. However, lorazepam did not influence eye-tracking data loss or peak velocity when it was corrected for saccadic amplitude (all *p* > 0.05; for full results, see Table 2).

**Table 1:** Descriptive statistics for effects of drug, saccade direction, and eccentricity on prosaccades

DV	PLC HF		PLC HN		PLC VF		PLC VN		LOR HF		LOR HN		LOR VF		LOR VN	
	*M*(*SD*)	α	*M*(*SD*)	α	*M*(*SD*)	α	*M*(*SD*)	α	*M*(*SD*)	α	*M*(*SD*)	α	*M*(*SD*)	α	*M*(*SD*)	α
Peak Velocity	296.91 (46.27)	0.88	206.56 (32.96)	0.89	261.27 (49.97)	0.94	181.71 (32.12)	0.84	288.84 (52.18)	0.84	195.28 (36.26)	0.91	254.65 (52.94)	0.82	169.06 (35.36)	0.90
Corrected Peak Velocity	41.47 (6.38)	0.90	57.75 (10.16)	0.93	38.67 (5.61)	0.85	52.48 (8.29)	0.88	41.07 (7.28)	0.60	56.82 (9.56)	0.95	38.73 (5.48)	0.80	51.83 (7.30)	0.77
Amplitude Gain	0.90 (0.06)	0.60	0.91 (0.09)	0.70	0.86 (0.07)	0.75	0.88 (0.08)	0.62	0.89 (0.09)	0.79	0.87 (0.10)	0.77	0.83 (0.09)	0.70	0.83 (0.13)	0.76
Latency	275.95 (21.22)	0.83	278.68 (22.86)	0.91	300.90 (23.30)	0.89	300.99 (24.14)	0.81	279.00 (17.86)	0.57	285.98 (21.34)	0.56	304.87 (21.30)	0.76	308.21 (22.05)	0.72
Data Loss	5.03 (9.02)	1.00	6.51 (14.33)	1.00	4.70 (10.47)	1.00	6.50 (15.96)	1.00	7.78 (12.04)	1.00	7.18 (8.42)	1.00	8.61 (10.97)	1.00	8.26 (13.52)	1.00

Numbers indicate the mean (standard deviation). α = Cronbach’s α. *PLC *Placebo, *LOR *Lorazepam, *HF *horizontal-far, *HN *horizontal-near, *VF *vertical-far, *VN *vertical-near. Measurement units: Peak velocity in °/s; corrected peak velocity in °/s by amplitude; amplitude gain in %/100; latency, saccade duration, and data loss in ms. Descriptive statistics for amplitude in ° can be found in the supplementary material, Table S1, for comparison to the free viewing task

**Table 2:** ANOVA results for all dependent variables in the prosaccade task for the three factors drug, direction, and eccentricity

DV	Drug	Direction	Eccentricity	Drug x Direction	Drug x Eccentricity	Direction x Eccentricity	Drug x Direction x Eccentricity
Peak Velocity	*F*_(1, 28)_ = 8.01, *p* = 0.008, $\eta$_p_^2^ = 0.223, CI [0.016,0.445]	*F*_(1, 28)_ = 54.81, *p* < 0.001, $\eta$_p_^2^ = 0.662, CI [0.416,0.773]	*F*_(1, 28)_ = 597.70, *p* < 0.001, $\eta$_p_^2^ = 0.955, CI [0.913,0.970]	*F*_(1, 28)_ = 0.00, *p* = 0.987, $\eta$_p_^2^ = 0.000, CI [0.000,1.000]	*F*_(1, 28)_ = 1.51, *p* = 0.229, $\eta$_p_^2^ = 0.051, CI [0.000,0.255]	*F*_(1, 28)_ = 4.75, *p* = 0.038, $\eta$_p_^2^ = 0.145, CI [0.000,0.371]	*F*_(1, 28)_ = 0.19, *p* = 0.670, $\eta$_p_^2^ = 0.007, CI [0.000,0.155]
Corrected Peak Velocity	*F*_(1, 28)_ = 1.05, *p* = 0.315, $\eta$_p_^2^ = 0.036, CI [0.000,0.230]	*F*_(1, 28)_ = 21.47, *p* < 0.001, $\eta$_p_^2^ = 0.434, CI [0.150,0.614]	*F*_(1, 28)_ = 306.11, *p* < 0.001, $\eta$_p_^2^ = 0.916, CI [0.839,0.944]	*F*_(1, 28)_ = 0.31, *p* = 0.581, $\eta$_p_^2^ = 0.011, CI [0.000,0.172]	*F*_(1, 28)_ = 0.82, *p* = 0.372, $\eta$_p_^2^ = 0.029, CI [0.000,0.216]	*F*_(1, 28)_ = 10.26, *p* = 0.003, $\eta$_p_^2^ = 0.268, CI [0.035,0.484]	*F*_(1, 28)_ = 0.03, *p* = 0.871, $\eta$_p_^2^ = 0.001, CI [0.000,0.100]
Amplitude Gain	*F*_(1, 28)_ = 6.63, *p* = 0.016, $\eta$_p_^2^ = 0.191, CI [0.006,0.416]	*F*_(1, 28)_ = 13.11, *p* = 0.001, $\eta$_p_^2^ = 0.319, CI [0.063,0.526]	*F*_(1, 28)_ = 0.01, *p* = 0.933, $\eta$_p_^2^ = 0.0000, CI [0.000,0.062]	*F*_(1, 28)_ = 1.13, *p* = 0.297, $\eta$_p_^2^ = 0.039, CI [0.000,0.235]	*F*_(1, 28)_ = 5.27, *p* = 0.029, $\eta$_p_^2^ = 0.158, CI [0.000,0.384]	*F*_(1, 28)_ = 2.13, *p* = 0.155, $\eta$_p_^2^ = 0.071, CI [0.000,0.283]	*F*_(1, 28)_ = 0.15, *p* = 0.704, $\eta$_p_^2^ = 0.005, CI [0.000,0.148]
Latency	*F*_(1, 28)_ = 6.73, *p* = 0.015, $\eta$_p_^2^ = 0.194, CI [0.007,0.419]	*F*_(1, 28)_ = 144.25, *p* < 0.001, $\eta$_p_^2^ = 0.837, CI [0.696,0.891]	*F*_(1, 28)_ = 4.43, *p* = 0.044, $\eta$_p_^2^ = 0.137, CI [0.000,0.362]	*F*_(1, 28)_ = 0.03, *p* = 0.866, $\eta$_p_^2^ = 0.001, CI [0.000,0.102]	*F*_(1, 28)_ = 4.24, *p* = 0.049, $\eta$_p_^2^ = 0.132, CI [0.000,0.356]	*F*_(1, 28)_ = 1.96, *p* = 0.172, $\eta$_p_^2^ = 0.066, CI [0.000,0.276]	*F*_(1, 28)_ = 0.08, *p* = 0.785, $\eta$_p_^2^ = 0.003, CI [0.000,0.128]
Data Loss	*F*_(1, 28)_ = 0.68, *p* = 0.415, $\eta$_p_^2^ = 0.024, CI [0.000,0.206]	*F*_(1, 28)_ = 0.85, *p* = 0.364, $\eta$_p_^2^ = 0.030, CI [0.000,0.218]	*F*_(1, 28)_ = 1.28, *p* = 0.267, $\eta$_p_^2^ = 0.044, CI [0.000,0.243]	*F*_(1, 28)_ = 2.69, *p* = 0.112, $\eta$_p_^2^ = 0.088, CI [0.000,0.306]	*F*_(1, 28)_ = 2.43, *p* = 0.130, $\eta$_p_^2^ = 0.080, CI [0.000,0.295]	*F*_(1, 28)_ = 0.10, *p* = 0.757, $\eta$_p_^2^ = 0.003, CI [0.000,0.136]	*F*_(1, 28)_ = 0.13, *p* = 0.720, $\eta$_p_^2^ = 0.005, CI [0.000,0.144]

There were significant interactions of drug x eccentricity for amplitude gain (*F*_(1, 28)_ = 5.27, *p* = 0.029, $\eta$_p_^2^ = 0.158, CI [0.000,0.384], Fig. 2b) and latency (*F*_(1, 28)_ = 4.24, *p* = 0.049, $\eta$_p_^2^ = 0.132, CI [0.000,0.356], Fig. 2c). *T*-tests indicated that lorazepam compared to placebo reduced amplitude gain only for near and not for far stimulus eccentricities (*t*(57) = -3.05, *p* = 0.012, *d* = -0.102, all other *p*_adj_. > 0.05). The drug x eccentricity interaction for latency showed that the increase in latency with lorazepam was numerically stronger for near than far targets, although *t*-tests indicated that no conditions differed significantly when *p*-values were adjusted (all *p*_adj_. > 0.05).

There were no other interactions involving the drug factor, indicating that lorazepam effects on peak velocity and amplitude were independent of eccentricity or direction and that the lorazepam effect on latency was independent of direction.

Main effects of direction showed that horizontal saccades had significantly higher peak velocities (*F*_(1, 28)_ = 54.81, *p* < 0.001, $\eta$_p_^2^ = 0.662, CI [0.416,0.773]), higher amplitude-corrected peak velocities (*F*_(1, 28)_ = 21.47, *p* < 0.001, $\eta$_p_^2^ = 0.434, CI [0.150,0.614]), larger amplitude gain (*F*_(1, 28)_ = 13.11, *p* = 0.001, $\eta$_p_^2^ = 0.319, CI [0.063,0.526]), and shorter latencies (*F*_(1, 28)_ = 144.25, *p* < 0.001, $\eta$_p_^2^ = 0.837, CI [0.696,0.891]) than vertical saccades, but there were no significant differences in eye-tracking data loss (*p* > 0.05, for full results, see Table 2).

Main effects of eccentricity showed that saccades to near stimulus locations had significantly lower saccadic peak velocities (*F*_(1, 28)_ = 597.70, *p* < 0.001, $\eta$_p_^2^ = 0.955, CI [0.913,0.970]) than those made to far stimulus locations. When peak velocity was corrected for saccadic amplitude, however, near stimulus eccentricities led to higher corrected peak velocities compared to far eccentricities (*F*_(1, 28)_ = 306.11, *p* < 0.001, $\eta$_p_^2^ = 0.916, CI [0.839,0.944]). Latencies were significantly higher (*F*_(1, 28)_ = 4.43, *p* = 0.044, $\eta$_p_^2^ = 0.137, CI [0.000,0.362]) for near than for far stimulus eccentricities. Stimulus eccentricity did not significantly influence eye-tracking data loss (*p* > 0.05, for full results, see Table 2).

Moreover, significant interaction effects of saccade direction x stimulus eccentricity were found on peak velocity (*F*_(1, 28)_ = 4.75, *p* = 0.038, $\eta$_p_^2^ = 0.145, CI [0.000,0.371]) and corrected peak velocity (*F*_(1, 28)_ = 10.26, *p* = 0.003, $\eta$_p_^2^ = 0.268, CI [0.035,0.484]). For both peak velocity and corrected peak velocity, *t*-tests showed that all comparisons differed significantly in all direction and eccentricity conditions (all *p*_adj_. < 0.001). Numerically, the effect of stimulus eccentricity on peak velocity as well as corrected peak velocity was larger for horizontal than for vertical saccades. For peak velocity, the effect of saccade direction was numerically larger in saccades made to far than to near stimuli. The opposite was true for corrected peak velocity. *T*-tests on the significant saccade direction x stimulus eccentricity interaction for amplitude gain indicated that the direction effect was only significant for far stimuli (*t*(57) = 5.65, *p* < 0.001, *d* = 0.176) but not for near ones (all other *p*_adj_. > 0.05). Further, the saccade direction x stimulus eccentricity interaction did not reach significance for latency or eye-tracking data loss (all *p* > 0.05, for full results, see Table 2).

### Free viewing task

Visual inspection of eye-tracking data led to the exclusion of two subjects from all analyses for the free viewing task, resulting in *n* = 28. For both excluded subjects, almost none of their fixations landed within the perimeter of the pictures. Descriptive results for the free viewing task are in Table 3.

**Table 3:** Descriptive statistics for effects of drug and saccade direction on saccades in free viewing

DV	PLC horizontal		PLC vertical		LOR horizontal		LOR vertical	
	*M* (*SD*)	α	*M* (*SD*)	α	*M* (*SD*)	α	*M* (*SD*)	α
Included Saccades	10.92 (2.27)	NA	11.12 (2.33)	NA	11.31 (1.80)	NA	11.26 (1.66)	NA
Peak Velocity	147.61 (31.20)	0.92	119.21 (18.40)	0.79	139.20 (29.74)	0.93	121.50 (27.81)	0.85
Corrected Peak Velocity	67.42 (12.08)	0.97	66.36 (8.29)	0.89	69.36 (16.68)	0.89	63.32 (9.17)	0.90
Amplitude	2.39 (0.32)	0.40	1.87 (0.20)	0.57	2.23 (0.41)	0.71	2.02 (0.46)	0.82
Saccade Frequency	1.13 (0.30)	0.83	0.58 (0.16)	0.68	1.16 (0.26)	0.70	0.59 (0.22)	0.85
Data Loss	3.77 (4.19)	0.97	3.73 (4.50)	0.90	4.25 (3.06)	0.97	4.11 (3.32)	0.96

Numbers indicate the mean (standard deviation). α = Cronbach’s α. *PLC *Placebo, *LOR *Lorazepam. Measurement units: Included saccades in absolute numbers per participant; peak velocity in °/s; corrected peak velocity in °/s by amplitude; amplitude in °; saccade frequency in mean per sec; data loss in ms

**Fig. 3:**
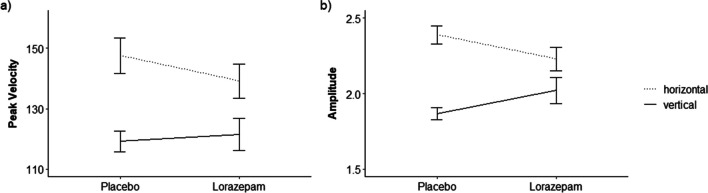
Interaction effects of drug condition and saccade direction in the free viewing task. *Note*. Error bars indicate standard error of the mean. **a** depicts the effect of drug condition and saccade direction on saccadic peak velocity in °/s, **b** on saccadic amplitude in °

ANOVAs revealed that the drug condition had no significant main effects on any of the dependent variables (all *p* > 0.05; for full ANOVA results for all dependent variables, see Table 4). However, there were significant drug x direction interactions (Fig. 3) on peak velocity (*F*_(1, 27)_ = 4.65, *p* = 0.040, $\eta$_p_^2^ = 0.147, CI [0.000,0.376]) and amplitude (*F*_(1, 27)_ = 11.82, *p* = 0.002, $\eta$_p_^2^ = 0.304, CI [0.051,0.517]). *T*-tests confirmed that for both peak velocity and amplitude, there were effects of direction (*peak velocity*: lorazepam, horizontal compared to vertical saccade direction: *t*(27) = 4.08, *p*_*adj.*_ = 0.001, *d* = 0.154, placebo, horizontal compared to vertical saccade direction *t*(27) = 7.74, *p*_*adj.*_ < 0.001, *d* = 0.286; *amplitude*: lorazepam, horizontal compared to vertical saccade direction: *t*(27) = 2.9, *p*_*adj.*_ = 0.028, *d* = 0.120, placebo, horizontal compared to vertical saccade direction *t*(27) = 8.52, *p*_*adj.*_ < 0.001, *d* = 0.494) but not drug condition (all *p*_*adj.*_ > 0.48) and showed that, numerically, the effect of saccade direction was less pronounced in the lorazepam than in the placebo condition. Conversely, lorazepam numerically reduced peak velocities compared to placebo, but only in horizontal saccades. Amplitude means (Table 3) showed that lorazepam reduced horizontal but increased vertical saccadic amplitude in comparison to placebo.

**Table 4:** ANOVA results for all dependent variables in the free viewing task for the factors drug and direction

DV	Drug	Direction	Drug x Direction
Peak Velocity	*F*_(1, 27)_ = 0.97, *p* = 0.333, $\eta$_p_^2^ = 0.035, CI [0.000,0.231]	*F*_(1, 27)_ = 53.25, *p* < 0.001, $\eta$_p_^2^ = 0.664, CI [0.412,0.776]	*F*_(1, 27)_ = 4.65, *p* = 0.040, $\eta$_p_^2^ = 0.147, CI [0.000,0.376]
Corrected Peak Velocity	*F*_(1, 27)_ = 0.08, *p* = 0.774, $\eta$_p_^2^ = 0.003, CI [0.000,0.135]	*F*_(1, 27)_ = 4.71, *p* = 0.039, $\eta$_p_^2^ = 0.149, CI [0.000,0.378]	*F*_(1, 27)_ = 2.00, *p* = 0.168, $\eta$_p_^2^ = 0.069, CI [0.000,0.285]
Amplitude	*F*_(1, 27)_ = 0.00, *p* = 0.960, $\eta$_p_^2^ = 0.000, CI [0.000,0.031]	*F*_(1, 27)_ = 55.37, *p* < 0.001, $\eta$_p_^2^ = 0.672, CI [0.424,0.782]	*F*_(1, 27)_ = 11.82, *p* = 0.002, $\eta$_p_^2^ = 0.304, CI [0.051,0.517]
Saccade Frequency	*F*_(1, 27)_ = 0.36, *p* = 0.553, $\eta$_p_^2^ = 0.013, CI [0.000,0.183]	*F*_(1, 27)_ = 155.77, *p* < 0.001, $\eta$_p_^2^ = 0.852, CI [0.718,0.902]	*F*_(1, 27)_ = 0.03, *p* = 0.854, $\eta$_p_^2^ = 0.001, CI [0.000,0.110]
Data Loss	*F*_(1, 27)_ = 0.38, *p* = 0.541, $\eta$_p_^2^ = 0.014, CI [0.000,0.185]	*F*_(1, 27)_ = 0.76, *p* = 0.390, $\eta$_p_^2^ = 0.028, CI [0.000,0.217]	*F*_(1, 27)_ = 0.34, *p* = 0.566, $\eta$_p_^2^ = 0.012, CI [0.000,0.180]

Saccade direction had a significant main effect on peak velocity (*F*_(1, 27)_ = 53.25, *p* < 0.001, $\eta$_p_^2^ = 0.664, CI [0.412,0.776]), amplitude (*F*_(1, 27)_ = 55.37, *p* < 0.001, $\eta$_p_^2^ = 0.672, CI [0.424,0.782]), peak velocity corrected for amplitude (*F*_(1, 27)_ = 4.71, *p* = 0.039, $\eta$_p_^2^ = 0.149, CI [0.000,0.378]), and saccade frequency (*F*_(1, 27)_ = 155.77, *p* < 0.001, $\eta$_p_^2^ = 0.852, CI [0.718,0.902]). Horizontal saccades were significantly faster, larger, and more frequent than vertical saccades.

Exploration patterns in the free viewing task were examined via a comparison of observed fixation maps to saliency maps predicted by the GBVS algorithm. After lorazepam administration, the mean correlation of fixation and saliency maps was *r* = 0.52 (*SD* = 0.15, max = 0.78, min = 0.15). For placebo assessments, the mean of correlations was *r* = 0.50 (*SD* = 0.13), with the maximum at *r* = 0.72 and the minimum at* r* = 0.15. The magnitude of these correlations indicates, on average, a moderate positive relationship between fixations and visual saliency. A *t*-test showed that there was no significant difference in correlations between lorazepam and placebo (*t*(39) = 1.26, *p* = 0.22, *d* = 0.398), indicating that GBVS maps did not predict fixation patterns better or worse as a function of drug condition. Thus, there was no evidence that people looked at different regions, or regions with different visual saliency, in the lorazepam condition.

### Subjective effects

Detailed results for subjective effects of lorazepam can be found in Faßbender et al. (2023). In summary, participants felt less alert and less content after lorazepam compared to placebo but experienced no differences in calmness or task load.

To examine whether subjective effects accompanied the objective effect of lorazepam on saccadic peak velocity, difference scores (placebo—lorazepam) were calculated for peak velocities in both tasks. Person’s correlations with alertness and contentedness showed no significant correlations (prosaccades: alertness: *r* = -0.168, *p* = 0.384, contentedness: *r* = 0.148, *p* = 0.442; free viewing: alertness: *r* = 0.051, *p* = 0.798, contentedness: *r* = -0.091, *p* = 0.645).

While participants were unable to reliably guess whether they had received placebo or lorazepam after the first assessment (*p* > 0.05), the proportion of participants (*n* = 25 out of 30) guessing the substance successfully at the end of the second assessment was significantly above chance level (*p* = 0.001).

## Discussion

The overall aim of the present study was to expand knowledge on the effects of benzodiazepines on saccadic eye movements. To do so, first, a prosaccade task was extended to include both vertical and horizontal saccades as well as different stimulus eccentricities. Second, lorazepam effects on saccade parameters were examined in a more naturalistic, free viewing task.

### Effects of lorazepam

The present results confirmed the decrease of horizontal saccadic peak velocities after lorazepam administration compared to placebo. Results also indicated that lorazepam effects on peak velocity did not affect horizontal and vertical prosaccades differently. On a neurophysiological level, this result suggests that excitatory input from superior colliculus which is reduced by lorazepam (Fuchs et al. 1985; Schiller et al. 1979) equally affects burst neurons in PPRF and riMLF. Importantly, the results further imply that lorazepam reduces peak velocity independent of saccade direction and stimulus eccentricity in this task. Taken together, these results substantiate the use of expanded prosaccade task designs in psychopharmacological research. They suggest that if saccadic peak velocity is reduced by a compound acting on the GABA_A_ receptor α1 subunit, thus likely exhibiting sedative side effects (Chen et al. 2014), peak velocity reductions will be discovered in simple prosaccade tasks independent of stimulus location. Moreover, the results also confirm the validity of horizontal prosaccade tasks to assess peak velocity as a biomarker of sedation.

Additionally, lorazepam reduced amplitude gains and increased latencies in the prosaccade task, even more so for near than far targets. These findings replicate previous studies and confirm that lorazepam causes a general destabilisation of saccadic control in prosaccade tasks, including spatial and temporal parameters (Ettinger et al. 2018).

Remarkably, however, the expected main effect of lorazepam on peak velocity did not emerge in the free viewing task. Further, lorazepam did not appear to have a direct, significant influence on any of the other saccadic parameters in the free viewing task. Correlations between GBVS maps and fixation patterns were, on average, moderately positive for both lorazepam and placebo but did not differ between drug conditions. The size of these correlations generally reproduced previous findings^5^ (Foulsham 2019; Kümmerer et al. 2015). Hence, as fixation patterns matched expectations, exploration behaviour, i.e., fixation patterns, was unaffected by lorazepam administration.

There are several possible explanations as to why there was no discernible main effect of lorazepam on saccadic eye movements during free viewing, despite the observed effects on prosaccades.

First, there are differences in instructions between the two tasks. In the prosaccade task, participants are instructed to look at the peripheral stimulus as soon as it appears and the stimulus is only visible for a short amount of time, which creates a speeded performance component that is not present during free viewing. This might cause detrimental lorazepam effects on peak velocity to emerge in prosaccade tasks but not during free viewing, especially as higher urgency in prosaccade tasks might lead to a general increase in peak velocity compared to free viewing where participants experience no such urgency (Montagnini and Chelazzi 2005).

Another possible explanation is that additional voluntary processes affect saccade generation (Foulsham 2019) during free viewing. Voluntary attentional processes are generally slower than reflex-like orienting (Müller and Rabbitt 1989), thus possibly slowing peak velocities during free viewing compared to prosaccades. The hypothesis of a ceiling effect on how much the firing rate of burst neurons, and therefore saccadic peak velocities, can be reduced (Galley 1989), could also play a role here. Generally lower peak velocities during free viewing could prevent additional decreases caused by lorazepam. Corrected peak velocities in the present study are, however, numerically lower in the prosaccade task than in the free viewing task, which would at least partially contradict these explanations. Still, the interactions of drug effect with saccade direction on peak velocity as well as saccade amplitude in the free viewing task could also be explained by this. In both interactions, the effect of saccade direction appeared to be reduced after lorazepam administration: Burst neurons for horizontal saccades might be affected by lorazepam, leading to reduced peak velocities and amplitudes, while for vertical saccades a ceiling in the reduction of burst neuron firing rate might be reached. This in turn could lead to the observation of a less pronounced effect of saccade direction on saccade parameters after lorazepam administration.

Reasons for the differences found in the influence of lorazepam on prosaccades versus on free viewing saccades may additionally lie in the underlying, diverse neurophysiology. A large network of cortical and subcortical areas is involved in saccade generation. Visual cortex in occipital lobe is activated by visual input (e.g., Hubel and Wiesel 1979). Signals from visual cortex reach PPRF and riMLF via superior colliculus (Collins et al. 2005). Parietal eye fields (PEF) are involved in triggering saccades, ending fixations, and shifting visual attention (Leigh and Zee 2015; Müri et al. 1996; Müri and Nyffeler 2008; Pierrot-Deseilligny et al. 1991). Frontal eye fields (FEF), supplementary eye fields (SEF), and dorsolateral prefrontal cortex (DLPFC) show higher activation during volitional than visually-guided saccades (O’Driscoll et al. 1995; Pierrot-Deseilligny et al. 1991; Rivaud et al. 1994). SEF support target selection in pre-learned saccade sequences and self-paced saccades (Petit et al. 1996), and DLPFC is more strongly involved in memory-guided saccades, response planning, and attentional control (Leigh and Zee 2015; McDowell et al. 2008; Müri et al. 1996). Within this network, most regions are more strongly activated during saccades that are generated while cognitive load is increased, for instance during anti-saccades that rely on inhibitory control or during free viewing where saccades are also influenced by endogenous attention and visual input is high (Pierce et al. 2019). Only activation in occipital cortex has been shown to be increased during prosaccades compared to saccades with comparatively more voluntary control (Clementz et al. 2010; Dyckman et al. 2007).

Those differences in saccade network activations could, to a certain extent, explain present findings. Likely higher engagement of FEF during free viewing might, at least in part, compensate for depression of excitatory signals from superior colliculus to brainstem saccade generators (Keller et al. 2008; Schiller et al. 1979; Scudder et al. 2002) that occurs after lorazepam administration. Differences in involvement of occipital cortex could also play a role. As only prosaccades but not free viewing saccades show detrimental lorazepam effects, the present results may indicate that occipital cortex is especially impacted by benzodiazepine administration. This is further supported by findings that implicated morphological variance in occipital regions in social anxiety disorder (Frick et al. 2014) and trait anxiety (Li et al. 2020). Moreover, patients with panic disorder were found to exhibit lower GABA levels in occipital lobe (Goddard et al. 2001; Schlegel et al. 1994).

### Effects of direction and eccentricity

Effects of saccade direction confirmed that horizontal saccades are seemingly ‘easier’ to produce, with higher peak velocities, larger amplitudes, and shorter latencies than vertical saccades (e.g., Becker and Jürgens 1990; Irving and Lillakas 2019). Burst neurons generating horizontal saccades are more densely distributed than burst neurons firing in order to produce vertical saccades (Büttner-Ennever 2008). This could, at least in part, explain systematic differences between horizontal and vertical saccades. Additionally, horizontal saccades might be generated more efficiently than vertical ones because synaptic connections might be stronger as horizontal eye movements are more common in natural conditions of the visual environment (Irving and Lillakas 2019).

In line with this, in free viewing, horizontal saccades are also more frequent (Bays and Husain 2012; Foulsham et al. 2011; Tatler and Vincent 2008). This might not only be due to the comparative ease of horizontal saccades but also because of what is called horizon bias (Foulsham et al. 2008): Horizontal edges are usually more prevalent than vertical ones and the horizon line is usually oriented horizontally in pictures used for free viewing tasks (and these pictures are often of landscape orientation). Thus, at least in picture-viewing experiments, horizontal saccades may be more common because of the distribution of potential saccade targets in the image.

Present findings concerning stimulus eccentricity effects on saccadic latency seemingly contradict results from Ettinger et al. (2018). They reported shorter latencies for near than for far stimuli, while the opposite was observed here. This discrepancy can be explained by the stimulus positions used in the two studies. Here, stimuli appeared 4° or 8° from the centre of the screen. Instead, in Ettinger et al. (2018), stimulus eccentricities were at 7.25° and 14.5°, therefore provoking much larger saccadic amplitudes. Interestingly, past studies found a U-shaped relationship between stimulus eccentricity and saccade latency, with shortest latencies at stimulus eccentricities between 3 to 9° (Kalesnykas and Hallett 1994; Smyrnis et al. 2002). This U-shaped relationship might explain the difference in findings between the two studies.

### Limitations

Due to picture sizes in the free viewing task and stimulus positions in the prosaccade task, the saccadic amplitudes provoked were systematically smaller in the free viewing than in the prosaccade task. As saccadic amplitudes were found to be related to image size in free viewing (von Wartburg et al. 2007), the informative value of a direct comparison of saccadic peak velocities in the two tasks is limited. The differences found might also be confounded by fatigue and boredom, as the absence of a drug effect during free viewing could be due to the task being more interesting and engaging than the prosaccade task. Also, time on task was shorter during free viewing than during prosaccades and stimuli differed between sessions in the free viewing task. Further, it should be noted that the number of pictures per category in the free viewing task was too small to merit a comparison of categories, therefore limiting possibilities to explain direction effects in more detail. Lastly, while the GBVS algorithm is a good feature-based saliency model, comparing fixation maps with GBVS maps might not be sensitive enough to uncover possible drug-induced changes in exploration patterns. Other saliency map models might be able to reveal more subtle differences.

## Conclusions and implications

To our knowledge, this is the first study to examine if the reduction in saccadic peak velocity after the administration of a benzodiazepine can also be found for vertical prosaccades and for saccades during free exploration of pictures. As the effect was not present during free viewing and lorazepam did not affect other saccadic parameters or exploration behaviour during free viewing, the question arises whether the real-life impacts of benzodiazepines’ sedative side effects have been overestimated by prosaccade tasks in the past. To address this question more thoroughly, further research is needed. Most importantly, it should be investigated what causes the lack of lorazepam effects during free viewing. Is it the lack of time pressure in comparison to the prosaccade task? Is it due to additional voluntary processes in free viewing saccades? Can controlling for picture categories or image content and properties explain findings? And finally, the influence of benzodiazepines on the neurophysiology of saccades will have to be examined closely to better gauge the extent of their sedative effects and underlying neural mechanisms.

## Supplementary Information

Below is the link to the electronic supplementary material.

Supplementary file1 (DOCX 237 KB)

## Data Availability

Data and analysis scripts can be found online in R Markdown format (https://osf.io/dr4qc/?view_only=a14dd494961a44208278c8c2ae54143a).

## Footnotes

1 Research questions regarding the free viewing task deviate from the preregistration for this study as the present paper’s focus lies on the types of saccades made, not the pictures viewed.

2 This includes the determination of saccade direction in relation to the fixation from which it originates: direction is determined as ‘left’ if the visual angle is greater than 135º or less than -135º, ‘right’ if the angle is between -45º and 45º, ‘up’ if the angle is between 45º and 135º, and ‘down’ if the angle is between -135º and -45º. Right and left saccade directions are summarised as ‘horizontal’, while ‘up’ and ‘down’ are summarised as ‘vertical’ saccades.

3 Perfect spatial accuracy of a saccade would be indicated by a gain of 1. Higher values indicate an overshooting, lower ones an undershooting saccade.

4 Data loss was calculated by dividing the number of blinks by their average duration in relation to the trial duration per participant and trial.

5 It should be noted that previous studies have indicated that bottom-up saliency overlaps with semantics and meaningful objects. The present study is not able to disentangle these influences on saccade guidance.
