# Supplementary material for: Effects of lorazepam on saccadic eye movements – evidence from prosaccade and free viewing tasks
Source: Psychopharmacology (Berl). 2024 Sep 3;242(2):271–84. doi: 10.1007/s00213-024-06672-z (PMC11775061; doi:10.1007/s00213-024-06672-z)
Supplement: Supplementary file 1 — Supplementary file1 (DOCX 237 KB) [file 213_2024_6672_MOESM1_ESM.docx]

Effects of Lorazepam on Saccadic Eye Movements – Evidence from Prosaccade and Free Viewing Tasks: Supplement

Philine M. Baumert^1^, Kaja Faßbender^1^, Maximilian W. M. Wintergerst^2, 5^, Jan H. Terheyden^2^, Behrem Aslan^3^, Tom Foulsham^4^, Wolf Harmening^2^ and Ulrich Ettinger^1^

^1^ Department of Psychology, University of Bonn, Bonn, Germany

^2^ Department of Ophthalmology, University of Bonn, Bonn, Germany

^3^ Department of Psychiatry and Psychotherapy, University of Bonn,

Bonn, Germany

^4^ Department of Psychology, University of Essex, UK

^5^ Augenzentrum Grischun, Chur, Switzerland

Effects of Lorazepam on Saccadic Eye Movements – Evidence from Prosaccade and Free Viewing Tasks: Supplement


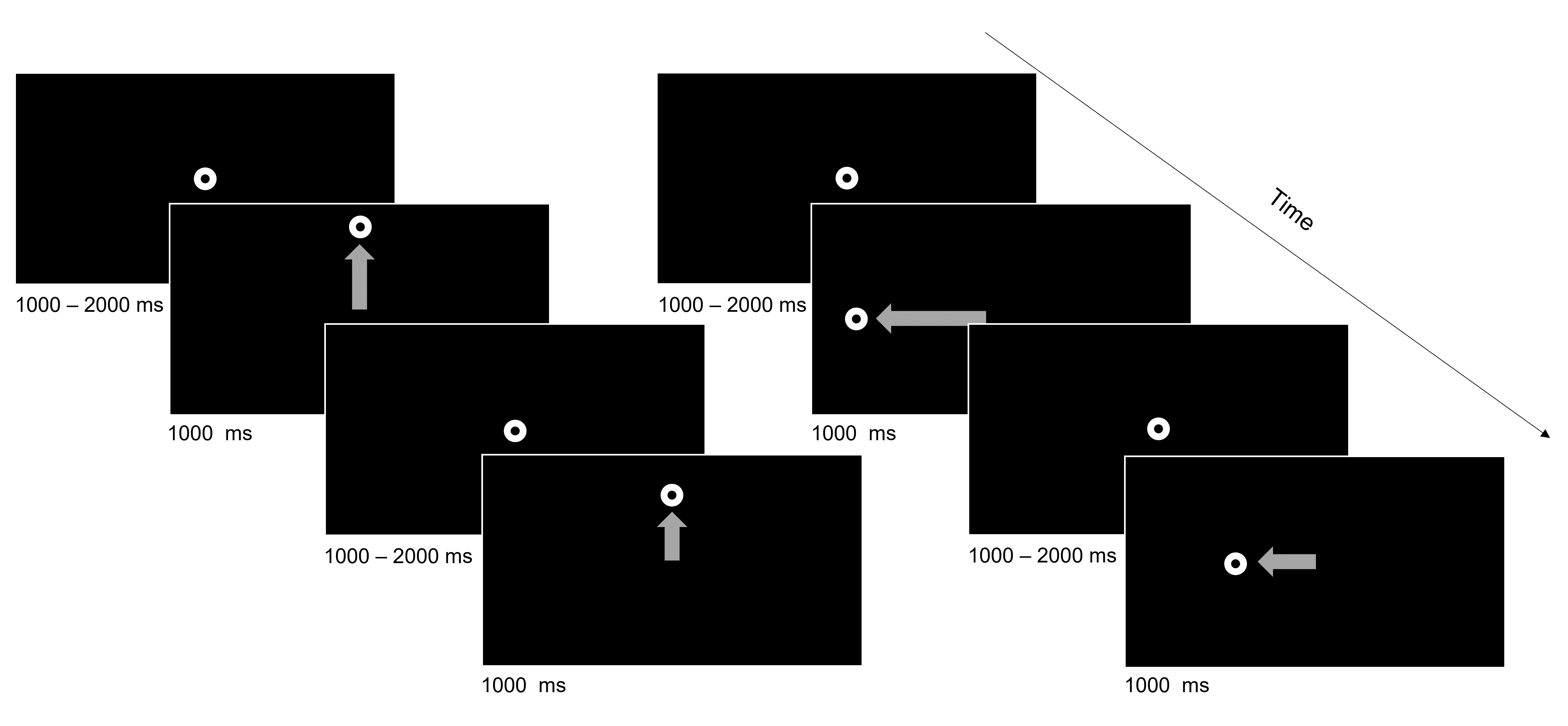
**Prosaccade Task**

*Note*. Depicted conditions are up-far, up-near, left-far, and left-near. Arrows indicated the direction of saccades made towards the peripheral stimulus. To improve visibility for demonstration purposes here, stimuli are larger in relation to the screen than they were in the original experiment.

**Fig. S1**Flowchart of the prosaccade task

**Free viewing task**

# IAPS Pictures

Included IAPS pictures were the following numbers (Lang et al., 2008):

- Animals: 1121, 1441, 1595, 1630, 1410, 1419, 1500, 1731
- Nature scenes: 5210, 5661, 5725, 7580, 5215, 5594, 5825, 5870
- Objects: 7011, 7012, 7062, 7078, 7001, 7014, 7052, 7081
- People: 2019, 2158, 2221, 2347, 2035, 2274, 2377, 2382
- Plants: 5010, 5201, 5202, 5532, 5001, 5030, 5040, 5500

**Fig. S2**
Flowchart of the free viewing task


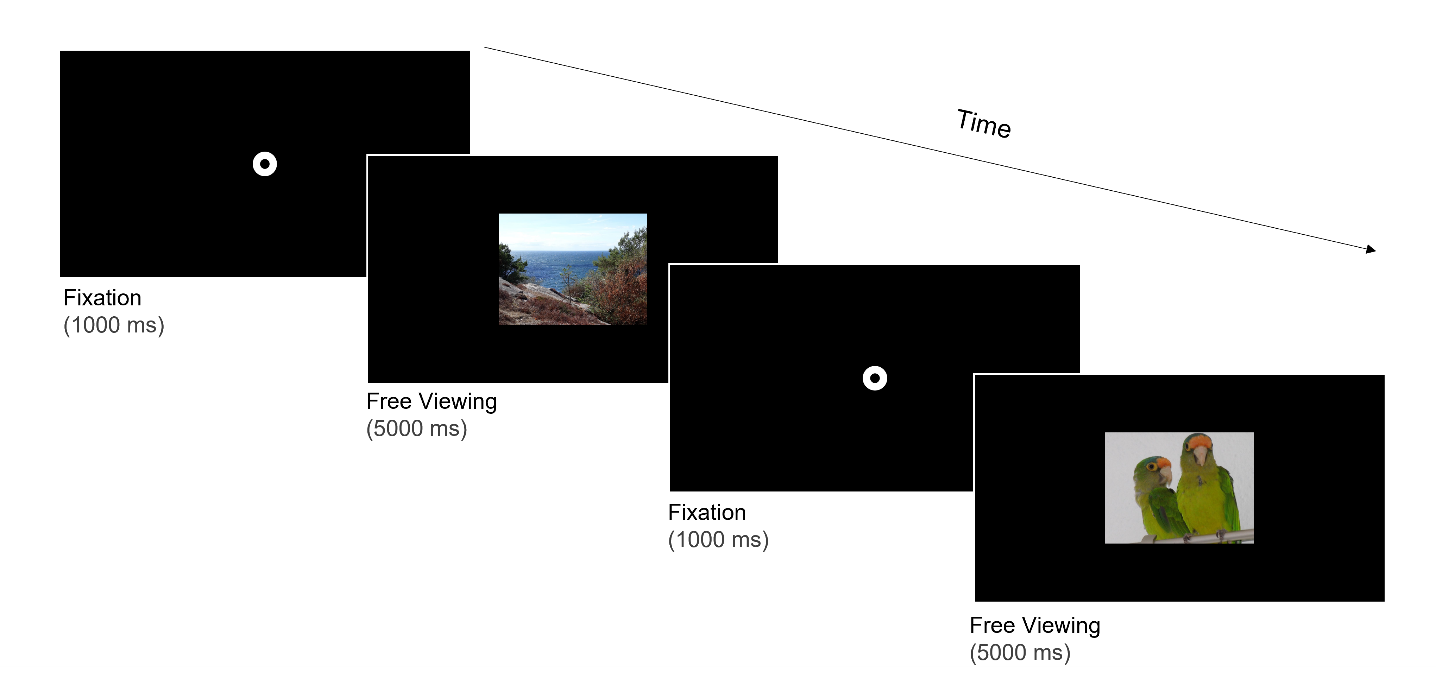


*Note.* Depicted pictures are exemplary and not part of the IAPS but from the first author’s personal archive. To improve visibility for demonstration purposes here, pictures as well as the fixation stimulus are larger in relation to the screen than they were in the original experiment.

**Empirical accuracy and precision of eye tracking data**

Empirical accuracy and precision of collected eye tracking data were calculated using the last fixation of the first and the last trial, separately for the prosaccade and the free viewing task. The eye-tracking data sample size per participant depended on the individual fixation duration (fixation duration x 1000). For each participant’s data sample per fixation, mean distances from the centre of the fixation circle were calculated in visual angle. Per participant, mean (for accuracy) and standard deviation (for precision) were calculated for X and Y coordinates and then combined using the Pythagorean theorem. In the prosaccade task, spatial accuracy ranged from 0.14° to 3.43° and was 1.27° on average, while spatial precision ranged between 0.06° and 0.38° with a mean of 0.15° in the first trial. In the last trial, accuracy ranged from 0.18° to 15.61° (although it should be noted here that the next highest value after the maximum was 3.97° and the outlier resulted from the participant in question closing their eyes for most of the trial) and had a mean of 1.43° and precision was between 0.04° and 0.77° with a mean of 0.19°. In the first trial of the free viewing task, accuracy had a minimum of 0.26° and a maximum of 3.47° with a mean of 1.48° and precision ranged from 0.04° to 0.34° with a mean of 0.14°. In the last trial, accuracy had a range from 0.08° to 5.08° with 1.99° on average, while precision ranged between 0.04° and 0.26° and yielded a mean of 0.13°.

# Cronbach’s alpha input information

In the prosaccade task, the following sample sizes were used to calculate Cronbach’s alpha:

- lorazepam, horizontal, near: included subjects: 29, included values per subject: 11
- lorazepam, horizontal, far: included subjects: 28, included values per subject: 11
- lorazepam, vertical, near: included subjects: 27, included values per subject: 10
- lorazepam, vertical, far: included subjects: 27, included values per subject: 13
- placebo, horizontal, near: included subjects: 29, included values per subject: 10
- placebo, horizontal, far: included subjects: 28, included values per subject: 15
- placebo, vertical, near: included subjects: 29, included values per subject: 10
- placebo, vertical, far: included subjects: 28, included values per subject: 19

In the free viewing task, the following sample sizes were used to calculate Cronbach’s alpha:

- lorazepam, horizontal: included subjects: 28, included values per subject: 19
- lorazepam, vertical: included subjects: 27, included values per subject: 10
- placebo, horizontal: included subjects: 28, included values per subject: 17
- placebo, vertical: included subjects: 28, included values per subject: 11

# Prosaccade task: Amplitude

Table S1. Descriptive statistics for effects of drug, saccade direction, and eccentricity on prosaccades.

| DV | PLC HF | PLC HN | PLC VF | PLC VN | LOR HF | LOR HN | LOR VF | LOR VN |
| --- | --- | --- | --- | --- | --- | --- | --- | --- |
| Amplitude | 7.23 (0.49) | 3.62 (0.34) | 6.86 (0.58) | 3.52 (0.32) | 7.16 (0.68) | 3.48 (0.41) | 6.64 (0.74) | 3.33 (0.54) |

*Note.* Note. Numbers indicate the mean (standard deviation). α = Cronbach’s α. PLC: Placebo, LOR: Lorazepam, HF: horizontal-far, HN: horizontal-near, VF: vertical-far, VN: vertical-near. Amplitude is given in °.

# Effects of order of drug administration in the prosaccade task

Table S2 a). Descriptive statistics for effects of drug and order of drug administration on data loss during the prosaccade task.

| DV | 1 PLC | 1 LOR | 2 PLC | 2 LOR |
| --- | --- | --- | --- | --- |
| Data Loss | 3.01 (4.37) | 10.71 (13.42) | 8.98 (17.75) | 4.57 (6.47) |

*Note.* Numbers indicate the mean (standard deviation). 1 indicates that the participant received placebo on the first assessment and 2 indicates that they were administered lorazepam on their first assessment. Data loss is given in ms.

*Table S2 b). Descriptive statistics for effects of drug, order of drug administration, and eccentricity on amplitude gain during the prosaccade task.*

| DV | 1 PLC far | 1 PLC near | 1 LOR far | 1 LOR near | 2 PLC far | 2 PLC near | 2 LOR far | 2 LOR near |
| --- | --- | --- | --- | --- | --- | --- | --- | --- |
| Amplitude Gain | 0.89 (0.06) | 0.89 (0.08) | 0.87 (0.10) | 0.86 (0.11) | 0.86 (0.08) | 0.90 (0.09) | 0.86 (0.09) | 0.84 (0.13) |

*Note.* Numbers indicate the mean (standard deviation). 1 indicates that the participant received placebo on the first assessment and 2 indicates that they were administered lorazepam on their first assessment.

Table S3. ANOVA results for the prosaccade task with between-subjects factor order.

| DV | Order | Order x drug | Order x drug x dir | Order x drug x ec | Order x drug x ec x dir |
| --- | --- | --- | --- | --- | --- |
| Amplitude Gain | *F*_(1, 27)_ = 0.28, *p* = .59, $\eta$_p_^2^ = .010,  CI [.0000,.174] | *F*_(1, 27)_ = 0.03, *p* = .873, $\eta$_p_^2^ = .00,  CI [.0000,.102] | *F*_(1, 27)_ = 1.19, *p* = .285, $\eta$_p_^2^ = .04,  CI [.0000,.244] | *F*_(1, 27)_ = 6.02, *p* = .021, $\eta$_p_^2^ = .18,  CI [.002,.411] | *F*_(1, 27)_ = 0.32, *p* = .579, $\eta$_p_^2^ = .01,  CI [.0000,.178] |
| Corr. Peak Velocity | *F*_(1, 27)_ = 1.72, *p* = .20, $\eta$_p_^2^ = .06,  CI [.0000,.272] | *F*_(1, 27)_ = 3.61, *p* = .068, $\eta$_p_^2^ = .11,  CI [.0000,.345] | *F*_(1, 27)_ = 0.99, *p* = .329, $\eta$_p_^2^ = .03,  CI [.0000,.232] | *F*_(1, 27)_ = 0.63, *p* = .435, $\eta$_p_^2^ = .02,  CI [.0000,.207] | *F*_(1, 27)_ = 2.08, *p* = .161, $\eta$_p_^2^ = .07,  CI [.0000,.288] |
| Data Loss | *F*_(1, 27)_ = 0.00, *p* = .98, $\eta$_p_^2^ = .0000,  CI [.0000,1.000] | *F*_(1, 27)_ = 5.58, *p* = .026, $\eta$_p_^2^ = .17,  CI [.0000,.400] | *F*_(1, 27)_ = 1.11, *p* = .301, $\eta$_p_^2^ = .04,  CI [.0000,.240] | *F*_(1, 27)_ = 1.61, *p* = .215, $\eta$_p_^2^ = .05,  CI [.0000,.267] | *F*_(1, 27)_ = 0.54, *p* = .467, $\eta$_p_^2^ = .02,  CI [.0000,.200] |
| Latency | *F*_(1, 27)_ = 0.83, *p* = .37, $\eta$_p_^2^ = .030,  CI [.0000,.222] | *F*_(1, 27)_ = 0.04, *p* = .841, $\eta$_p_^2^ = .00,  CI [.0000,.115] | *F*_(1, 27)_ = 2.00, *p* = .169, $\eta$_p_^2^ = .06,  CI [.0000,.285] | *F*_(1, 27)_ = 0.18, *p* = .678, $\eta$_p_^2^ = .00,  CI [.0000,.157] | *F*_(1, 27)_ = 0.07, *p* = .788, $\eta$_p_^2^ = .00,  CI [.0000,.131] |
| Peak Velocity | *F*_(1, 27)_ = 1.21, *p* = .28, $\eta$_p_^2^ = .043,  CI [.0000,.245] | *F*_(1, 27)_ = 2.30, *p* = .141, $\eta$_p_^2^ = .07,  CI [.0000,.297] | *F*_(1, 27)_ = 0.23, *p* = .638, $\eta$_p_^2^ = .00,  CI [.0000,.166] | *F*_(1, 27)_ = 3.23, *p* = .084, $\eta$_p_^2^ = .10,  CI [.0000,.332] | *F*_(1, 27)_ = 0.76, *p* = .391, $\eta$_p_^2^ = .02,  CI [.0000,.217] |

*Note.* Results for all dependent variables in the prosaccade task for between subject factor order, that indicates which substance was administered first, as well as its possible interactions with the three within-subjects factors drug, direction (dir), and eccentricity (ec). Measurement units: Peak velocity in °/s; corrected peak velocity in °/s by amplitude; latency and data loss in ms.

# Effects of order of drug administration in the free viewing task

Table S4. ANOVA results for the free viewing task.

| DV | Order | Order x drug | Order x drug x direction |
| --- | --- | --- | --- |
| Amplitude | *F*_(1, 26)_ = 1.27, *p* = .270, $\eta$_p_^2^ = .047,  CI [.0000,.256] | *F*_(1, 26)_ = 1.88, *p* = .182, $\eta$_p_^2^ = .068,  CI [.0000,.287] | *F*_(1, 26)_ = 0.00, *p* = .967,  $\eta$_p_^2^ = .0000,  CI [.0000,.020] |
| Corrected Peak Velocity | *F*_(1, 26)_ = 0.00, *p* = .953, $\eta$_p_^2^ = .0000,  CI [.0000,.043] | *F*_(1, 26)_ = 0.02, *p* = .886, $\eta$_p_^2^ = .001,  CI [.0000,.098] | *F*_(1, 26)_ = 1.86,  *p* = .185,  $\eta$_p_^2^ = .067,  CI [.0000,.285] |
| Data Loss | *F*_(1, 26)_ = 0.02, *p* = .902, $\eta$_p_^2^ = .001,  CI [.0000,.089] | *F*_(1, 26)_ = 0.07, *p* = .789, $\eta$_p_^2^ = .003,  CI [.0000,.135] | *F*_(1, 26)_ = 2.16,  *p* = .153,  $\eta$_p_^2^ = .077,  CI [.0000,.299] |
| Peak Velocity | *F*_(1, 26)_ = 0.36, *p* = .553, $\eta$_p_^2^ = .014,  CI [.0000,.188] | *F*_(1, 26)_ = 2.38, *p* = .135, $\eta$_p_^2^ = .084,  CI [.0000,.308] | *F*_(1, 26)_ = 0.75,  *p* = .393, $\eta$_p_^2^ = .028,  CI [.0000,.223] |
| Saccade Frequency | *F*_(1, 26)_ = 0.01, *p* = .905, $\eta$_p_^2^ = .001,  CI [.0000,.087] | *F*_(1, 26)_ = 4.02, *p* = .055, $\eta$_p_^2^ = .134,  CI [.0000,.366] | *F*_(1, 26)_ = 0.02,  *p* = .879, $\eta$_p_^2^ = .001,  CI [.0000,.102] |

*Note.* Results for all dependent variables in the free viewing task for the two within subject factors drug and direction, as well as the between subject factor order, that indicates which substance was administered first. Measurement units: Peak velocity in °/s; corrected peak velocity in °/s by amplitude; amplitude in °; data loss in ms.

# Saliency

Table S5. GBVS map correlation.

| Image | Category | Placebo | Lorazepam |
| --- | --- | --- | --- |
| 1121_gr.png | animal | 0.71 | 0.73 |
| 1410_gr.png | animal | 0.72 | 0.63 |
| 1419_gr.png | animal | 0.60 | 0.61 |
| 1441_gr.png | animal | 0.59 | 0.45 |
| 1500_gr.png | animal | 0.39 | 0.25 |
| 1595_gr.png | animal | 0.54 | 0.76 |
| 1630_gr.png | animal | 0.60 | 0.61 |
| 1731_gr.png | animal | 0.15 | 0.15 |
| 2019_gr.png | people | 0.47 | 0.63 |
| 2035_gr.png | people | 0.33 | 0.41 |
| 2158_gr.png | people | 0.60 | 0.58 |
| 2221_gr.png | people | 0.69 | 0.78 |
| 2274_gr.png | people | 0.64 | 0.63 |
| 2347_gr.png | people | 0.44 | 0.37 |
| 2377_gr.png | people | 0.49 | 0.53 |
| 2382_gr.png | people | 0.23 | 0.22 |
| 5001_gr.png | plants | 0.59 | 0.52 |
| 5010_gr.png | plants | 0.50 | 0.51 |
| 5030_gr.png | plants | 0.56 | 0.55 |
| 5040_gr.png | plants | 0.40 | 0.26 |
| 5201_gr.png | plants | 0.32 | 0.35 |
| 5202_gr.png | plants | 0.50 | 0.55 |
| 5210_gr.png | nature | 0.71 | 0.76 |
| 5215_gr.png | nature | 0.44 | 0.52 |
| 5500_gr.png | plants | 0.55 | 0.57 |
| 5532_gr.png | plants | 0.50 | 0.57 |
| 5594_gr.png | nature | 0.60 | 0.60 |
| 5661_gr.png | nature | 0.66 | 0.67 |
| 5725_gr.png | nature | 0.30 | 0.31 |
| 5825_gr.png | nature | 0.28 | 0.33 |
| 5870_gr.png | nature | 0.45 | 0.47 |
| 7001_gr.png | objects | 0.44 | 0.34 |
| 7011_gr.png | objects | 0.45 | 0.64 |
| 7012_gr.png | objects | 0.50 | 0.58 |
| 7014_gr.png | objects | 0.46 | 0.42 |
| 7052_gr.png | objects | 0.43 | 0.55 |
| 7062_gr.png | objects | 0.49 | 0.57 |
| 7078_gr.png | objects | 0.56 | 0.50 |
| 7081_gr.png | objects | 0.63 | 0.61 |
| 7580_gr.png | nature | 0.62 | 0.66 |

*Note.* Correlations were calculated for each picture in the free viewing task dependent on the drug condition.
